# The association between epigenetic ageing from childhood to early adulthood and psychotic-like experiences in early adulthood

**DOI:** 10.1017/S003329172510055X

**Published:** 2025-06-30

**Authors:** Zoe Hart, Anna Großbach, Andrew J Simpkin, Esther Walton

**Affiliations:** 1 Department of Psychology, University of Bath, Bath, UK; 2 School of Mathematical and Statistical Sciences, University of Galway, Galway, Ireland

**Keywords:** ALSPAC, DNA methylation, epigenetic age, psychosis, longitudinal

## Abstract

**Background:**

Psychotic-like experiences (PLEs) are associated with cognitive impairment and premature mortality, which may be indicative of accelerated biological ageing. Epigenetic clocks provide a measure of biological age based on DNA methylation, yet the long-term relationship between epigenetic ageing and PLEs remains largely unclear. We tested the relationship between epigenetic ageing and PLEs using a 17-year longitudinal approach.

**Methods:**

Epigenetic ageing was calculated using four epigenetic clocks (DunedinPACE, Cortical EpiAge, Horvath, and PCGrimAge) in a sample from the Avon Longitudinal Study of Parents and Children (ALSPAC), a large population-based birth cohort (*n* = 1840, 56.8% females). We modeled epigenetic ageing from up to three repeated measures collected between ages 7 and 24 using a linear mixed-effects model to calculate (1) average epigenetic age [mean-centered intercept] and (2) rate of epigenetic ageing over this 17-year period [slope]. We then compared these two measures between individuals who developed PLEs in early adulthood (*n* = 95) against those who did not (*n* = 1745).

**Results:**

Results showed that a faster rate (slope) of longitudinal PCGrimAge was predictive of PLEs (OR = 1.79, 95% CI [1.13–2.85], *p* = .014), although this association was no longer significant after adjusting for smoking. There was a non-significant effect in the same direction for other clocks. Average epigenetic age (mean-centered intercept) was not associated with PLEs.

**Conclusions:**

Our findings suggest that the observed association between accelerated rate of epigenetic ageing, measured with PCGrimAge, from childhood to early adulthood, and the development of PLEs in early adulthood may be explained by smoking.

## Background

Psychotic-like experiences (PLEs) feature hallucinations, delusions and experiences of thought interference; they occur in the general population with an estimated prevalence of around 7.8% (Kelleher & Cannon, 2011; McGrath et al., 2015) and often start in early adulthood with a median age of onset of 26 years (McGrath et al., 2016). Although PLEs are associated with an up to fourfold increased risk of psychotic disorder (Healy et al., 2019), they differ from more clinical forms of psychosis in that individuals might not experience distress and therefore might not engage in help-seeking behaviors (Os et al., 2009). Risk factors, including prenatal, biological, and environmental stressors, may contribute to the development of PLEs in early adulthood (Staines et al., 2024; Turley, Drake, Killackey, & Yung, 2019). There is a need to understand the developmental pathways to PLEs in order to develop predictive markers of risk.

DNA methylation (DNAm) is an epigenetic modification, involving the addition of methyl groups to DNA, primarily at cytosine bases (Ooi, O’Donnell, & Bestor, 2009). Changes in DNAm are common throughout development and ageing and can lead to alterations in gene expression, ultimately affecting biological pathways underpinning developmental processes. PLEs have previously been linked to DNAm at multiple stages of development (Roberts et al., 2019), highlighting the importance of investigating epigenetic biomarkers for PLE risk identification. Epigenetic age, an age estimate derived from DNAm states (Horvath, 2013), is a commonly studied epigenetic biomarker associated with adverse health outcomes (Marioni et al., 2015). Numerous epigenetic clocks – often categorized into first-, second-, and third-generation clocks – have been developed that are trained on different populations and ageing biomarkers (Belsky et al., 2022; Lu et al., 2019). Therefore, they may measure different aspects of the life course ageing process.

First-generation epigenetic clocks, including Horvath’s pan-tissue clock (Horvath, 2013), were developed from DNAm patterns associated with chronological age. Horvath’s clock was trained on 51 healthy tissues and cell types. While performing well in brain tissue, this clock was not specifically designed for brain-associated traits such as PLEs. Cortical EpiAge, although also a first-generation clock and trained on chronological age, was developed using cortex tissue samples (*n* = 1047, age range = 1–107 years, median = 57 years; (Shireby et al., 2020). Its tissue-specificity may make it a good estimator of changes in epigenetic ageing associated with brain-associated traits such as PLEs, partially due to neuroimaging evidence for altered cortical changes during development in those who transition to psychosis (Ziermans et al., 2010). However, first-generation clocks may be trained too closely on chronological age and may not offer much insight into factors beyond age that may impact biological ageing. Second-generation epigenetic clocks incorporate health- and age-related measures, including biomarkers of mortality, health span, and smoking. An example is GrimAge, trained on surrogate biomarkers of plasma proteins and smoking in pack-years to predict lifespan (Higgins-Chen et al., 2022; Lu et al., 2019). Third-generation clocks are measures of pace of ageing. DunedinPACE was developed from 19 biomarkers measured longitudinally assessing different physiological systems (Belsky et al., 2022). It can predict an individual’s pace of ageing from a single blood sample. In summary, these three generations of clocks differ in the way they were developed and hence measure different aspects of the life course ageing process, of which some may relate to PLEs.

Epigenetic age research on PLEs in the general population is scarce. Several studies have focused mainly on clinical populations and reported on the cross-sectional association between epigenetic age and psychotic disorders (mainly schizophrenia). For example, Dada et al. (2021) investigated the relationship between two epigenetic age measures and psychosis severity in 138 individuals with schizophrenia spectrum disorders (mean age of 44.1 years). Epigenetic age acceleration using two first-generation clocks (Horvath and Hannum) was positively correlated with psychosis severity. Talarico et al. (2021) explored epigenetic age (Horvath) in 60 first-episode psychosis patients (mean age of 25.9 years) and 60 healthy controls (mean age of 28.8 years). The patient group displayed a lower average epigenetic age than the healthy controls, suggesting psychosis could be associated with deceleration of epigenetic age. However, both studies were cross-sectional, focused on clinical populations, and only included first-generation clocks, providing limited insight into the longitudinal relationships between PLEs and epigenetic age in the general population and across later-generation clocks.

Iftimovici et al. (2022) implemented a longitudinal approach to investigate the association between epigenetic age and transition to psychosis. They recruited 38 help-seeking participants (i.e. ultra-high risk of psychosis; mean age 20.5–23.0 years) and estimated their epigenetic age at baseline and at a 1-year follow-up using the Horvath clock, comparing converters to psychosis with non-converters. At baseline, they found that epigenetic age acceleration was reduced in future converters compared to non-converters. However, at the 1-year follow-up, there was no longer evidence for a difference in mean epigenetic age acceleration between the two groups. These findings suggest that between baseline and follow-up, epigenetic ageing of the future converters may have accelerated, eliminating the difference between groups. Longitudinal research could therefore capture differences in epigenetic ageing that cross-sectional research cannot measure, but to which degree this relationship can be observed in non-help-seeking individuals with PLEs from the general population is unclear.

**Figure 1.**
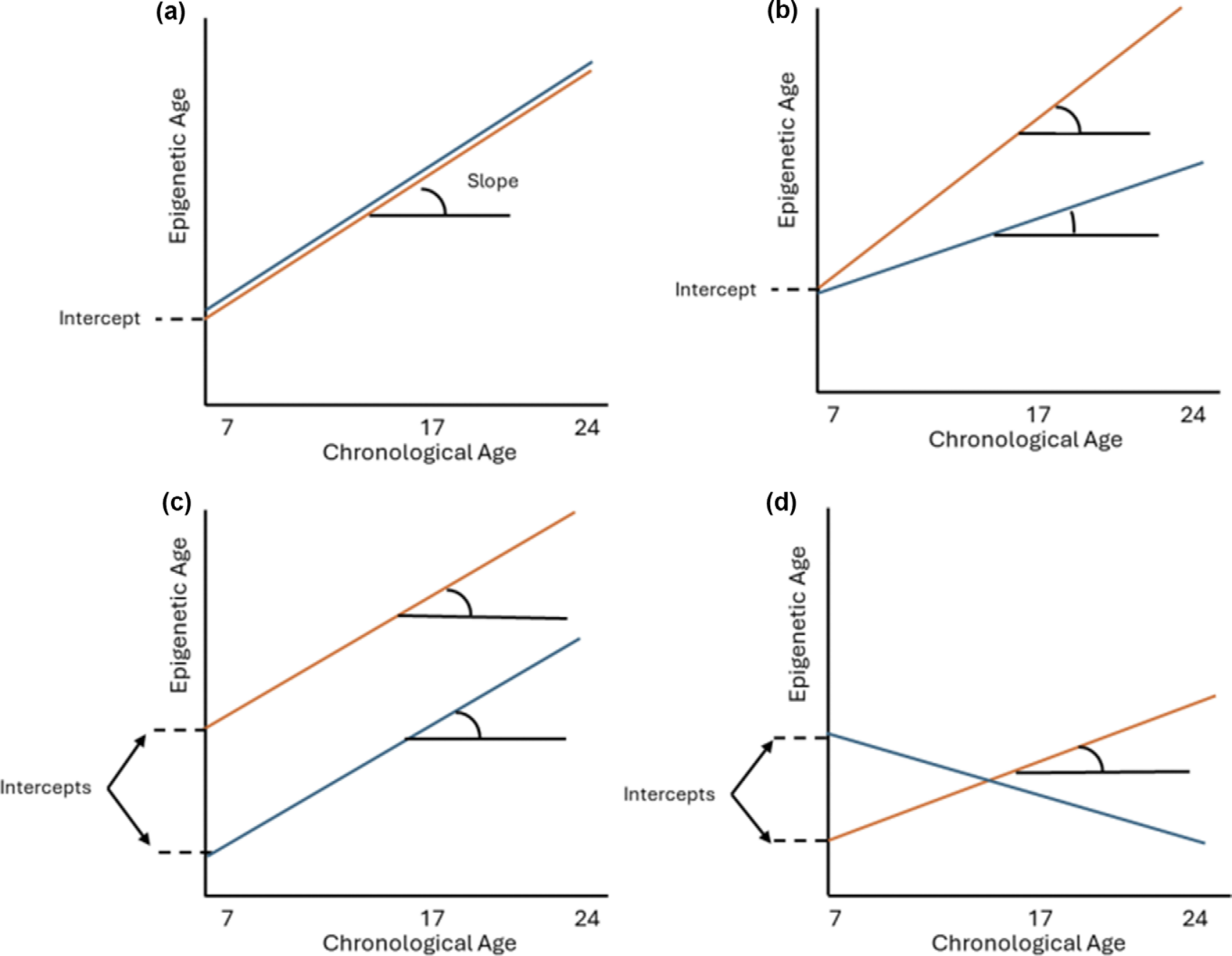
The random intercept represents the average epigenetic *age* across the dataset from 7 to 24 years, while the random slope represents epigenetic *ageing* (i.e. change) from 7 to 24 years. The panels show differences between average epigenetic age (random intercept) and epigenetic ageing from 7 to 24 years (random slope), between two groups (in orange and blue). (a) No difference between average epigenetic age and epigenetic ageing. (b) Difference in epigenetic ageing (slope) between the two groups but no difference in average epigenetic age (intercept). (c) Difference in average epigenetic age (intercept) between two groups but no difference in epigenetic ageing (slope). (d) Average epigenetic age (intercept) and epigenetic ageing (slope) are both different between the two groups.

The present study builds upon previous research by following a sample of 1840 population-based, non-help-seeking individuals longitudinally from 7 to 24 years, to investigate whether epigenetic ageing over multiple time points from childhood to early adulthood could predict the likelihood of developing PLEs measured in early adulthood (~ age 24 years). We used four clocks covering all three generations (Horvath, Cortical EpiAge, GrimAge [principal component, PCGrimAge], and DunedinPACE) and linear mixed-effects models (LMMs) to generate random intercepts (representative of average epigenetic *age*) and slopes (representative of epigenetic *ageing*) for each participant. Figure 1 visualizes differences between average epigenetic *age* (i.e. the intercept) and epigenetic *ageing* over time (i.e. the slope). Random intercepts and slopes were included as predictors in a logistic general linear model to investigate whether average epigenetic age (across ages 7, 17, and 24 years) and epigenetic ageing (from 7 to 24 years) were associated with PLEs in early adulthood. We hypothesized faster epigenetic ageing from 7 to 24 years (with weaker effects for epigenetic age), especially for second- and third-generation clocks, in individuals who developed PLEs at 24 years compared to those who did not.

## Methods

### Sample

This analysis used data from the Avon Longitudinal Study of Parents and Children (ALSPAC), a population-based cohort (Boyd et al., 2013; Fraser et al., 2013; Northstone et al., 2019). Further information on recruitment and ethical approval is presented in Supplementary Section 1. In this study, we analyzed data from three time points: childhood (~7 years), adolescence (~17 years), and early adulthood (~24 years). For inclusion, subjects were required to have DNAm data for at least one of the three time points as well as PLE data at 24 years. This resulted in a sample of 1840 individuals. The longitudinal overlap of the DNAm sample, divided into those with psychotic-like symptoms (PLIKS, cases) and without (controls), is presented in Supplementary Figure 1.

### Epigenetic age

DNAm data were obtained from whole-blood samples at 7, 17, and 24 years using the Illumina Infinium HumanMethylation450 BeadChip array or the Illumina MethylationEPIC array. DNAm preprocessing was performed using the meffil package in R, as previously described (Min et al., 2018). Blood-based epigenetic age was calculated using four clocks (further described in Supplementary Table 1): Cortical EpiAge, DunedinPACE, Horvath, and PCGrimAge, as described previously (Belsky et al., 2022; Horvath, 2013; Lu et al., 2019; Shireby et al., 2020.

### Covariates

The analyses consisted of a primary analysis and a secondary analysis adjusted for smoking. Both analyses included covariates of sex, age, estimated cell-type proportions, and array type. Cell type proportions were estimated using the Houseman reference panel and included B cells, CD4T, CD8T, natural killer cells, monocytes, and granulocytes (Houseman et al., 2016). Inspection of data revealed significant negative correlations between CD4T and granulocytes at every time point (7 years, *r* = −0.82; 17 years, *r* = −0.80; 24 years, *r* = −0.78; Supplementary Figures 2, 3, and 4). CD4T cells were therefore removed from the analysis to reduce multicollinearity. A secondary analysis was run, additionally adjusting for smoking using a measure of epigenetically predicted smoking status (Philibert et al., 2020), as smoking is known to be associated with both DNAm signatures and psychosis (Mustonen et al., 2018; Shenker et al., 2013). The epigenetic marker of smoking was methylation at site cg05575921 linked to the gene AHRR, ranging between 0 and 1. A lower value indicates a higher degree of smoking.

### Psychotic-like symptoms

At 24 years, participants from the ALSPAC cohort were assessed for PLEs in the previous 6 months via semi-structured interviews to assess PLIKS (Horwood et al., 2008). The interview consists of 12 core questions covering hallucinations, delusions, and thought disturbance, adapted from existing diagnostic interviews (S. Zammit et al., 2008). Raters scored PLIKS as not present, suspected, or definite, which were defined as not being caused by sleep or fever. Only young people who had attended a PLIKS interview at 24 years were included in the analysis. For this study, PLIKs were used as a binary outcome, either ‘none’ or ‘suspected/definite’ for at least one of the 12 core questions.

### Statistical analysis

The analyses were carried out in two stages: LMMs to estimate intercepts and slopes of epigenetic age for every individual, followed by logistic regression models with estimated random intercepts and slopes modeled as predictors and PLEs as the outcome (Supplementary Figure 5). All analyses were performed using R v4.3.2 using the nlme package.

In the first stage, LMMs were used to estimate random intercepts (average epigenetic age across time points) and slopes (longitudinal epigenetic ageing trajectories from childhood to early adulthood) for each individual. Chronological age was modeled as the predictor, and epigenetic age was modeled as the outcome. The models controlled for sex, array, and five cell type composition scores (B cells, CD8T, granulocytes, monocytes, and natural killer cells) and included random effects of participant ID and chronological age for three clocks (PCGrimAge, Cortical EpiAge, and Horvath). For DunedinPACE, only ID was included as a random effect, due to little change in pace of ageing between time points (i.e. DunedinPACE is a rate measure of ageing, and this rate remained fairly consistent over time). A secondary analysis was adjusted additionally for tobacco smoking, and a methylation measure of smoking was included as a fixed effect in the LMM. From the LMM, participant-specific random intercepts were generated for all four clocks as well as random slopes for Horvath, PCGrimAge, and Cortical EpiAge, using best linear unbiased predictions. The random intercept summarizes the average epigenetic age for the participant across all three time points (7, 17, and 24 years), and the random slope summarizes each individual’s epigenetic ageing trajectory from 7 to 24 years (Figure 1).

In the second stage, the individual random intercept and random slope coefficients for each clock were included as predictors in a logistic regression model with PLIKS as the outcome (i.e. four models), to investigate whether average epigenetic age (random intercept) or epigenetic ageing (random slope) predicted the likelihood of developing PLEs in early adulthood.

## Results

### Sample characteristics

The sample consisted of 1840 individuals with a higher proportion of females (57%) and those of White ethnicity (96%, Table 1). Of the 1840 individuals, 95 (5.2%) experienced PLEs at 24 years. Those with PLEs were more likely to smoke, were less likely to study at university, and came from a lower socio-economic background compared to those without PLEs. The mean age at methylation data collection at each time point was 7.44 (7 years), 17.6 (17 years), and 24.4 (24 years, Supplementary Table 2). Epigenetic age using Horvath, Cortical EpiAge, and PCGrimAge clocks increased across the time points and was consistently greater than chronological age (Supplementary Figure 6; Supplementary Table 2). Mean pace of epigenetic ageing (DunedinPACE) remained stable across the three time points at 0.84, 0.84, and 0.87. A LMM was fitted to the data to capture individual variations in epigenetic age and ageing. Chronological age was significantly associated with epigenetic age for all clocks, with epigenetic age increasing for every year of chronological age using Horvath, Cortical EpiAge, and PCGrimAge clocks (Horvath, *β* = 0.78, 95% CI [0.74–0.82], *p* < .001; Cortical EpiAge, *β* = 0.82, 95% CI [0.79–0.85], *p* < .001; PCGrimAge, *β* = 0.84, 95% CI [0.83–0.86], *p* < .001; DunedinPACE, *β* = 0.00, 95% CI [0.00–0.00], *p* = .029; Supplementary Table 3). Epigenetic ageing from 7 to 24 years of the 95 individuals with PLEs and a random sample of 95 controls are shown in spaghetti plots in Supplementary Figure 6.

**Table 1.** Sample description

Characteristic	Overall, N = 1,840^1^	No PLEs, N = 1,745^1^	PLEs, N = 95^1^	*p*-Value^2^
Gender				0.7
Male	794/1,840 (43%)	751/1,745 (43%)	43/95 (45%)	
Female	1,046/1,840 (57%)	994/1,745 (57%)	52/95 (55%)	
Ethnicity				0.8
White	1,643/1,710 (96%)	1,559/1,624 (96%)	84/86 (98%)	
Non-White	67/1,710 (3.9%)	65/1,624 (4.0%)	2/86 (2.3%)	
Missing	130	121	9	
Smoking				<0.001
No	406/772 (53%)	396/734 (54%)	10/38 (26%)	
Yes	366/772 (47%)	338/734 (46%)	28/38 (74%)	
Missing	1,068	1,011	57	
Education (university)				0.005
No	316/1,312 (24%)	295/1,250 (24%)	21/62 (34%)	
Still at university	75/1,312 (5.7%)	67/1,250 (5.4%)	8/62 (13%)	
Yes	921/1,312 (70%)	888/1,250 (71%)	33/62 (53%)	
Missing	528	495	33	
Maternal education				0.011
Other secondary education	812/1,732 (47%)	757/1,643 (46%)	55/89 (62%)	
A level	533/1,732 (31%)	516/1,643 (31%)	17/89 (19%)	
University degree	387/1,732 (22%)	370/1,643 (23%)	17/89 (19%)	
Missing	108	102	6	
Number of PLEs				
Mean (range)	1.7 (1.0, 7.0)	NA	1.7 (1.0, 7.0)	
Missing	1,745	1,745	0	

1 *n*/N (%).

2 Pearson’s chi-squared test; Fisher’s exact test.

### Primary analysis

#### Epigenetic age (intercept)–PLEs

**Figure 2.**
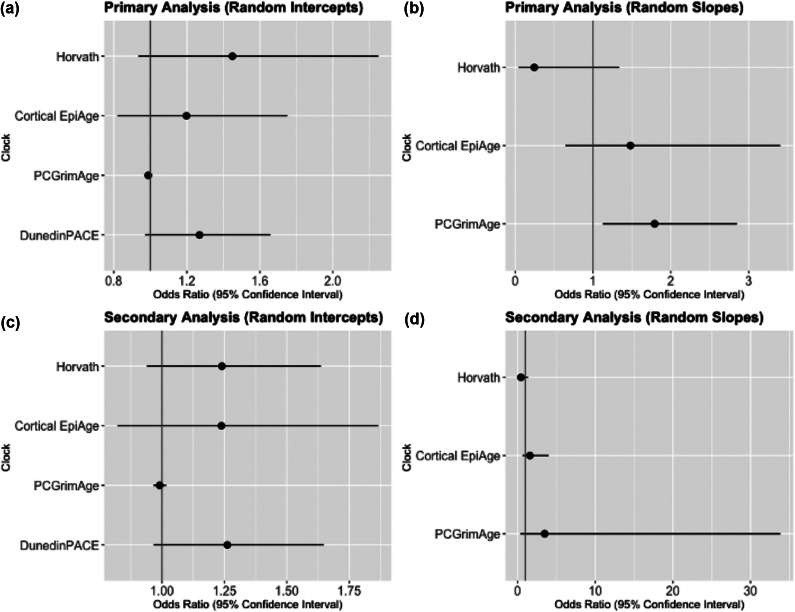
Forest plots of the odds ratios of PLEs at 24 years for an increase in epigenetic age (intercept) and ageing (slope) with 95% confidence intervals for average epigenetic age (random intercepts, a and c) and epigenetic ageing (random slopes, b and d) from the primary (top panel) and secondary analyses, additionally adjusting for smoking (bottom panel).

Average epigenetic age (i.e. the random intercept) was overall not associated with PLEs with any epigenetic clock (Figure 2). In our study sample, we found that those with a higher average epigenetic age (using Horvath and Cortical EpiAge clocks) appeared to have greater odds of PLEs at 24 years, but these associations were not significant (Horvath, OR = 1.45, *p* = .097, 95% CI [0.93–2.25]; Cortical EpiAge, OR = 1.2, *p* = .351, 95% CI [0.82–1.75], Table 2). There was also only a very small, non-significant association between average epigenetic age, using PCGrimAge, and PLEs at 24 years (OR = 0.99, *p* = .267, 95% CI [0.97–1.01], Table 2). Those with a greater average pace of epigenetic ageing, measured using DunedinPACE, appeared to have slightly greater odds of PLEs at 24 years. However, due to wide confidence intervals, this association was also non-significant (OR = 1.27, *p* = .080, 95% CI [0.97–1.66], Table 2).

**Table 2.** Results of logistic regression with estimated random intercepts and slopes modeled as predictors and PLEs as the outcome, controlling for sex, array, and cell type

Primary analysis		Odds ratio	CI (95%)	*p*-Value
Epigenetic age (intercept)	Horvath	1.45	0.93–2.25	0.097
	Cortical EpiAge	1.2	0.82–1.75	0.351
	PCGrimAge	0.99	0.97–1.01	0.267
	DunedinPACE	1.27	0.97–1.66	0.08
Epigenetic ageing (slope)	Horvath	0.25	0.05–1.34	0.104
	Cortical EpiAge	1.48	0.64–3.41	0.355
	PCGrimAge	1.79	1.13–2.85	0.014*

*Note*: CI, confidence intervals.

* Significant after Bonferroni-correction for three tests (*p*-value = 0.05/3 = 0.016).

#### Epigenetic ageing (slope)–PLEs

A faster increase in epigenetic ageing (i.e. the random slope), measured with PCGrimAge, from 7 to 24 years was significantly associated with a 79% greater likelihood of PLEs at 24 years (OR = 1.79, *p* = .014, 95% CI [1.13–2.85], Table 2). In contrast, those with faster epigenetic ageing from 7 to 24 years (using Horvath clock) appeared to have slightly reduced odds of PLEs at 24 years, but this reduction was non-significant (OR = 0.25, *p* = .104, 95% CI [0.05–1.34] (Table 2)). Those with faster epigenetic ageing from 7 to 24 years using Cortical EpiAge appeared to be at greater odds of developing PLEs at 24 years, but again this difference was also non-significant (OR = 1.48, *p* = .355, 95% CI [0.64–3.41], Table 2).

### Secondary analysis (additionally adjusted for smoking)

#### Chronological age–epigenetic age

When the models were additionally adjusted for epigenetically predicted smoking behavior, chronological age remained significantly associated with epigenetic age (Horvath, *β* = 0.79, 95% CI [0.75–0.82], *p* < .001; Cortical EpiAge, *β* = 0.81, 95% CI [0.78–0.84], *p* < .001; PCGrimAge, *β* = 0.82, 95% CI [0.81–0.83], *p* < .001; DunedinPACE, *β* = 0.00, 95% CI [0.00–0.00], *p* = .028; Supplementary Table 4), evidencing that the epigenetic age–chronological age association was not driven by smoking behavior.

#### Epigenetic age (intercept)–PLEs

After adjusting for smoking, there remained no overall associations between average epigenetic age and PLEs with any epigenetic clock (Figure 2). In our study sample, we found that those with a higher average epigenetic age (using Horvath and Cortical EpiAge clocks) appeared to have greater odds of PLEs at 24 years, but these were not significant associations (Horvath, OR = 1.24, *p* = .129, 95% CI [0.94–1.64]; Cortical EpiAge, OR = 1.24, *p* = .308, 95% CI [0.82–1.87], Table 3). There remained a very small, non-significant association between average epigenetic age and PLEs at 24 years using PCGrimAge (OR = 0.99, *p* = .467, 95% CI [0.97–1.02], Table 3). In our sample, those with a greater average pace of epigenetic ageing, measured using DunedinPACE, appeared to have greater odds of PLEs at 24 years, but this difference was non-significant (OR = 1.26, *p* = .088, 95% CI [0.97–1.65], Table 3).

**Table 3.** Results of logistic regression with estimated random intercepts and slopes modeled as predictors and PLEs as the outcome, controlling for sex, array, cell type, and smoking

Secondary analysis		Odds ratio	CI (95%)	*p*-Value
Epigenetic age (intercept)	Horvath	1.24	0.94–1.64	0.129
	Cortical EpiAge	1.24	0.82–1.87	0.308
	PCGrimAge	0.99	0.97–1.02	0.467
	DunedinPACE	1.26	0.97–1.65	0.088
Epigenetic ageing (slope)	Horvath	0.43	0.14–1.36	0.150
	Cortical EpiAge	1.58	0.63–3.98	0.329
	PCGrimAge	3.48	0.36–33.83	0.282

*Note*: CI, confidence intervals.

#### Epigenetic ageing (slope)–PLEs

In the secondary analysis, there was no longer a significant association between epigenetic ageing, measured with PCGrimAge, and PLE likelihood (OR 3.48, *p* = 0.282, 95% CI [0.36–33.83], Table 3). Those with faster epigenetic ageing (Horvath clock) appeared to have reduced odds of PLEs at 24 years, but this was a non-significant reduction (OR = 0.43, *p* = .150, 95% CI [0.14–1.36], Table 3). Those with faster epigenetic ageing from 7 to 24 years using Cortical EpiAge appeared to be at greater odds of developing PLEs at 24 years, but this was a non-significant association (OR = 1.58, *p* = .329, 95% CI [0.63–3.98], Table 3).

## Discussion

This study investigated whether epigenetic ageing from childhood to early adulthood is a predictor of PLEs in early adulthood, using a sample of 1840 individuals from ALSPAC, a longitudinal population-based birth cohort study. We utilized four clocks (Horvath, Cortical EpiAge, PCGrimAge, and DunedinPACE) and LMMs to estimate two measures: average epigenetic *age* (intercept) and epigenetic *ageing* (slope) at three time points (7, 17, and 24 years of age). We found that faster epigenetic *ageing* measured with PCGrimAge (but not with Horvath and Cortical EpiAge) predicted PLEs at 24 years; however, this association was no longer significant after adjusting for smoking. Average epigenetic *age* (Horvath, Cortical EpiAge, and PCGrimAge) or pace of ageing (DunedinPACE) was not associated with PLEs.

We did not find an association between average epigenetic *age* and PLEs in our sample, contradicting findings from clinical studies. For example, Talarico et al. (2021) found reduced epigenetic age (using the Horvath clock) in first-episode psychosis patients compared to controls. Dada et al. (2021) reported epigenetic age acceleration to be associated with psychosis severity in patients with schizophrenia spectrum disorders. These conflicting results could be explained by differences in the study populations. Previous studies utilized clinical samples with a greater range of ages and higher mean age. For example, Dada et al. (2021) had a clinical study sample with a mean age of 44.1 years, SD = 11.98. In our study, ‘cases’ encompassed population-based individuals in their early twenties with sub-clinical PLIKS. It is possible that alterations in epigenetic age only emerge later during disease progression or for traits closer to clinical measures of psychosis. This also aligns with related research showing that brain age acceleration, another measure of biological age, is greater in patients than in individuals at risk (Koutsouleris et al., 2014).

Faster epigenetic *ageing* measured with PCGrimAge predicted a greater likelihood of PLEs at 24 years, although this association was partially explained by smoking. This finding suggests that the change in epigenetic age over time (i.e. ageing, rather than average epigenetic age) may be a more sensitive measure in the context of PLE risk. Our study aligns with results from a previous longitudinal study (Iftimovici et al., 2022), which followed participants for 1 year. The authors reported that epigenetic ageing of future converters to psychosis accelerated compared to non-converters over a 1-year period. Similarly to our results, their effects were no longer significant after controlling for smoking, although smoking was not associated with epigenetic age gap at baseline nor with epigenetic age acceleration over this 1-year follow-up period. Importantly, we expanded these results by focusing on epigenetic ageing from childhood through early adulthood. Our findings suggest that faster epigenetic ageing in the context of PLEs may already start in childhood and develop over a 12-year period into early adulthood.

It is possible that our findings reflect changes in environmental influences (such as smoking initiation) over time. For example, recent research has suggested that most of the variance in within-individual changes in epigenetic ageing across a two-year period and about half of the between-individual differences were attributable to unique environmental factors (Kuznetsov et al., 2024). This highlights the potential importance of environmental influences on longitudinal epigenetic ageing. Future studies need to apply stronger causal designs to study whether and which environmental factors could alter epigenetic ageing trajectories.

For example, the association between epigenetic ageing and PLEs attenuated when adjusting for smoking, suggesting that our findings could be partially driven by smoking. Uptake of smoking during the period studied may have led to an acceleration of epigenetic ageing, as smoking can leave a strong signature in DNAm at particular CpG sites (Zeilinger et al., 2013) and is also associated with increased epigenetic age acceleration (Gao, Zhang, Breitling, & Brenner, 2016). Increased cigarette smoking is also associated with increased odds of PLEs, in a dose–response manner (Bhavsar et al., 2017). Therefore, it is possible that epigenetic ageing in PLEs might be impacted by smoking. Future studies are needed to explore whether smoking reduction could be a potential avenue for intervention to slow down epigenetic ageing in people at risk of psychosis.

We found differential clock effects, specifically for PCGrimAge, with no association for Horvath, Cortical EpiAge, or DunedinPACE. This contradicts Iftimovici et al. Iftimovici et al. (2022), who found epigenetic ageing acceleration in converters to psychosis, using the Horvath clock. The lack of replication using Horvath could be due to differences in the follow-up period. Our study followed participants from 7 to 24 years, whereas Iftimovici et al. followed participants aged 20–23 years over a 1-year period. It is possible that our study was less sensitive to detect short-term epigenetic ageing alterations specifically in early adulthood and instead better placed to measure long-term changes in epigenetic ageing from childhood to early adulthood. The specificity of our findings to PCGrimAge could also be due to the health-related measures PCGrimAge is trained on. PCGrimAge has been shown to be strongly associated with mortality (Lu et al., 2019), and PLEs have also been linked to higher mortality and reduced life expectancy (Sharifi et al., 2015). Therefore, PCGrimAge acceleration may reflect DNAm changes in those with PLEs that could be associated with a greater risk of mortality.

Strengths of our study include (1) our choice of multiple clocks from multiple generations chosen based upon their tissue relevance and links to adverse health outcomes; (2) a substantial follow-up period of 17 years, while previous studies were either cross-sectional or followed participants for only a couple of years; and (3) the focus on sub-threshold PLIKS in the general population.

Our study also needs to be considered in light of the following limitations. Despite its large overall sample size (*n* = 1840), our study could have benefited from a larger sample of those who experienced PLEs at 24 years (*n* = 95) to increase power. Furthermore, PLE is only one risk factor for psychosis, and many people with PLEs never transition to psychosis at all, especially those who made only one such experience, did not feel distressed, did not show functional impairments (Os et al., 2009; Sullivan et al., 2020; Stanley Zammit et al., 2013). In our sample, 64% reported only one PLE, and a previous study on ALSPAC participants with PLEs at 18 years reported low rates of professional help-seeking behaviors (12.8%, Zammit et al., 2013). In addition, we did not consider other risk factors such as genetic risk and family history of psychosis. Therefore, we recommend being cautious when applying our results to the clinical context of psychosis. To control for smoking, we used an epigenetic proxy of smoking, which is only an indirect measure of smoking behavior. We also did not control for other possible confounders that are known to influence the risk of PLEs (e.g. genetic risk, trauma, prenatal stress). Lastly, our study recruited participants from Avon, a former county in the UK, with 95.7% of our sample from a White ethnic background. Ethnicity may influence PLE risk (Morgan et al., 2009, *p.* 201), and so further research is warranted focusing on different ethnicities to explore potential differences in findings.

## Conclusions

Overall, our study provides important insights into epigenetic ageing over time and its potential associations with PLEs. We found (1) that epigenetic ageing from childhood to early adulthood may be a better predictor of PLEs in early adulthood than average epigenetic age and (2) that this association is partially explained by smoking. This paves the way for future studies extending our research, following participants over an even greater time period to further understand how PLE-linked epigenetic ageing and related lifestyle behaviors such as smoking are associated with transitioning to psychotic disorder.

## Supporting information

Hart et al. supplementary material Hart et al. supplementary material

## Data Availability

ALSPAC data access is through a system of managed open access. The steps below highlight how to apply for access to the data included in the data note and all other ALSPAC data: (1) Please read the ALSPAC access policy (http://www.bristol.ac.uk/media-library/sites/alspac/documents/researchers/data-access/ALSPAC_Access_Policy.pdf), which describes the process of accessing the data and samples in detail and outlines the costs associated with doing so. (2) You may also find it useful to browse our fully searchable research proposals database (https://proposals.epi.bristol.ac.uk/?q=proposalSummaries), which lists all research projects that have been approved since April 2011. Please submit your research proposal (https://proposals.epi.bristol.ac.uk/) for consideration by the ALSPAC Executive Committee. You will receive a response within 10 working days to advise you whether your proposal has been approved.

## Supplementary material

The supplementary material for this article can be found at https://doi.org/10.1017/S003329172510055X.
