# Supplementary material for: The association between epigenetic ageing from childhood to early adulthood and psychotic-like experiences in early adulthood
Source: Psychol Med. 2025 Jun 30;55:e182. doi: 10.1017/S003329172510055X (PMC13040582; doi:10.1017/S003329172510055X)
Supplement: Hart et al. supplementary material [file S003329172510055Xsup001.docx]

**Supplementary Materials for:**

**The association between epigenetic ageing from childhood to early adulthood with psychotic-like experiences in early adulthood**

**SM Methods**

**SM 1.1 Sample**

Pregnant women resident in Avon, UK with expected dates of delivery between 1st April 1991 and 31st December 1992 were invited to take part in the study (*N* = 14 541 pregnancies; 13 988 infants were alive at 1 year (Boyd et al., 2013; Fraser et al., 2013; Northstone et al., 2019). Further recruitment of eligible cases resulted in a sample of 15 447 pregnancies, of which 14 901 infants were alive at 1 year of age. Please note that the study website contains details of all the data that is available through a fully searchable data dictionary and variable search tool <https://www.bristol.ac.uk/alspac/researchers/our-data/>.

The total sample size for analyses using any data collected after the age of seven is 15,447 pregnancies, of these 14,901 children were alive at 1 year of age. Participants were followed over time from birth. For a sub sample of 1840 individuals. DNAm was measured as part of the Accessible Resource for Integrated Epigenomic Studies (ARIES) (Relton et al., 2015). Ethical approvals are in place for all sources of biological samples and data in ARIES in accordance with the Declaration of Helsinki. Consent for biological samples has been collected in accordance with the Human Tissue Act (2004). We analysed data from three time points: childhood (~ age 7), adolescence (~ age 17), and early adulthood (~ age 24). For inclusion, subjects were required to have DNAm data for at least one of the three time points as well as PLEs data at age 24. This resulted in a sample of 1840 individuals. The longitudinal overlap of the DNAm sample, divided into those with psychotic-like symptoms (cases) and without (controls), is presented in Supplementary Figure 1.

Ethical approval for the study was obtained from the ALSPAC Ethics and Law Committee and the Local Research Ethics Committees. Informed consent for the use of data collected via questionnaires and clinics was obtained from participants, following the recommendations of the ALSPAC Ethics and Law Committee at the time. Study data after 2014 were collected with REDCap (Research Electronic Data CAPture tools; (Harris et al., 2009)); a secure web application for online data collection, hosted at the University of Bristol (<https://catalyst.harvard.edu/redcap/>).

**
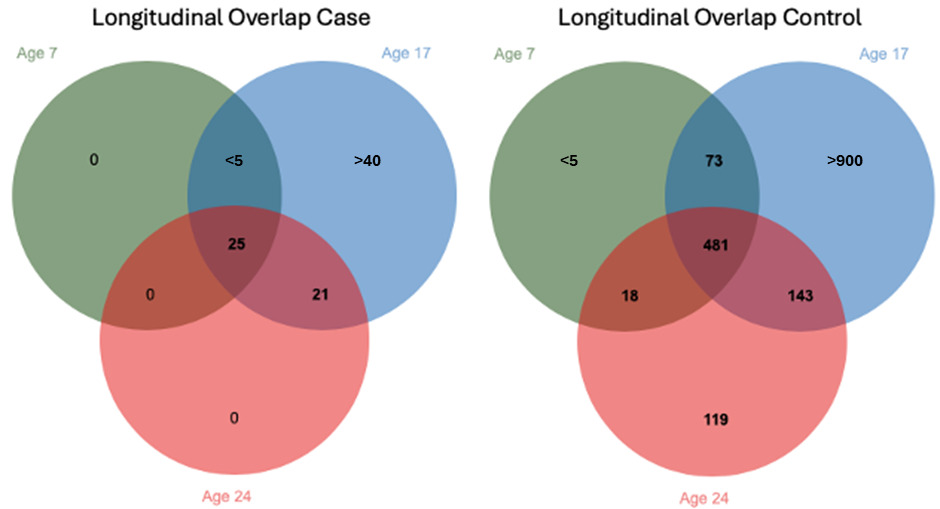
** **Supplementary Figure 1.** Longitudinal overlap of total DNAm sample at ages 7, 17, and 24 in individuals with psychotic-like symptoms at age 24 and controls. Where needed, approximate cell sizes are shown to ensure that exact cell sizes <5 (which may contain zero) cannot be recovered from other information provided in this figure, in line with ALSPAC requirements.

**SM 1.2 Epigenetic Age**

| Denotation | Reference | Trained phenotype | CpG Sites | Tissues Derived |
| --- | --- | --- | --- | --- |
| Horvath | (Horvath, 2013) | Chronological age | 353 | 51 Tissues |
| Cortical EpiAge | (Shireby et al., 2020) | Chronological age | 347 | Cortex |
| PCGrimAge | (Lu et al., 2019) | Mortality | 513 | Whole Blood |
| DunedinPACE | (Belsky et al., 2022) | 19 biomarkers assessing cardiovascular, metabolic, renal, hepatic, immune, dental, and pulmonary systems | 173 | Whole Blood |

**Supplementary Table 1.**Epigenetic clocks used in this study.

**SM 1.3 Covariance tables per time point**


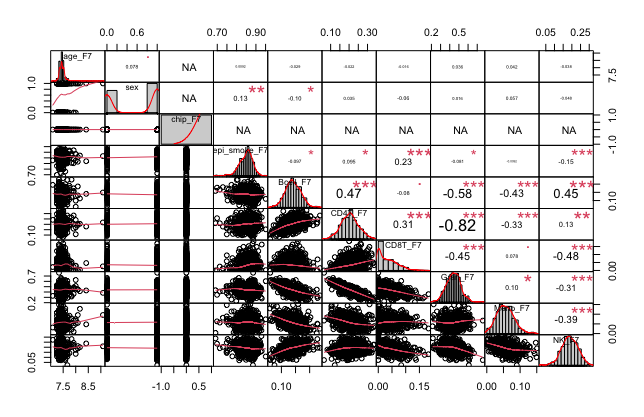


**Supplementary Figure 2.** Covariance table at age 7 highlights high correlation between covariates, particularly CD4T and granulocytes.


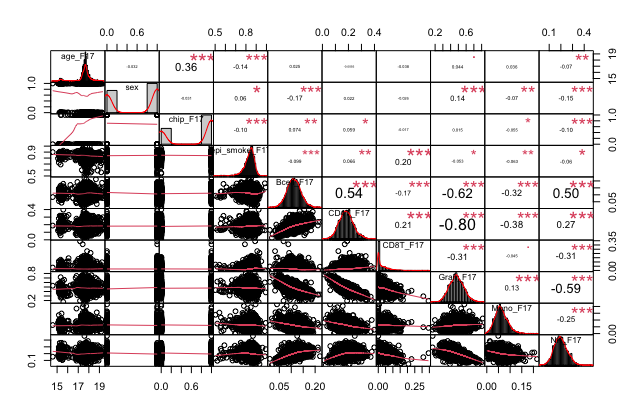


**Supplementary Figure 3.** Covariance table at age 17 highlights high correlation between covariates, particularly CD4T and granulocytes.


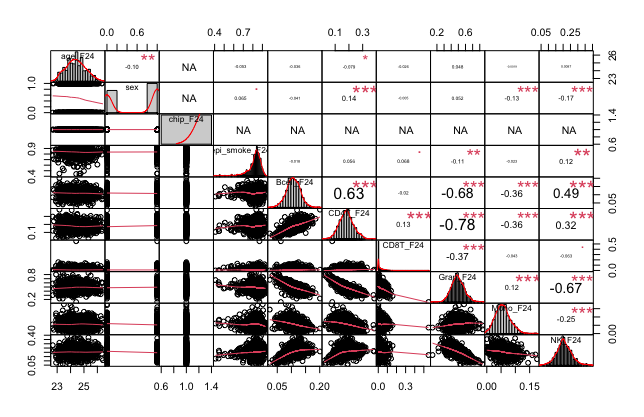
**Supplementary Figure 4.** Covariance table at age 24. High correlation between CD4T and granulocytes here and at age 7 and 17 justifies out decision to remove CD4T cells from analyses to reduce multicollinearity.

**SM 1.4 Statistical analysis**


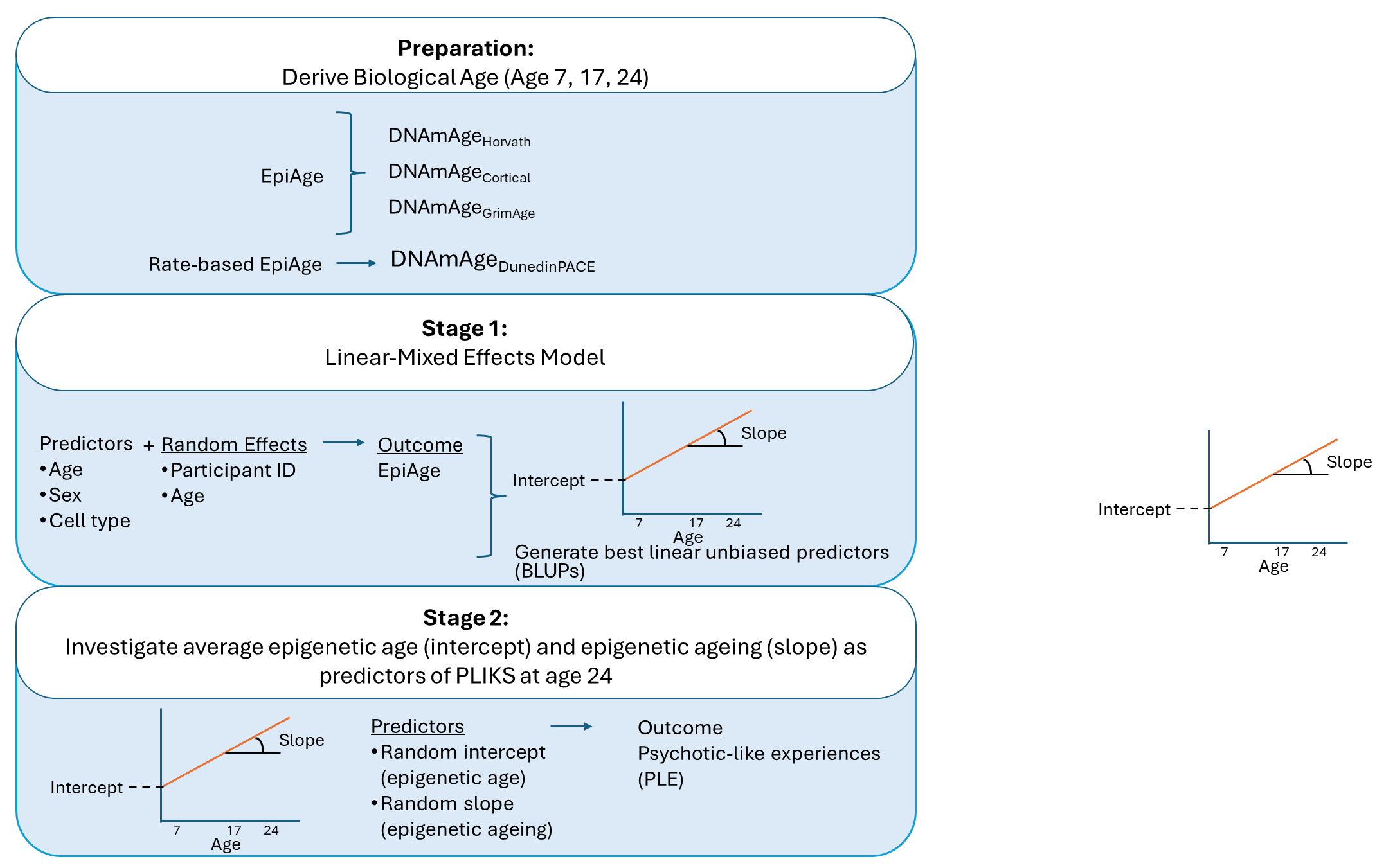


**Supplementary Figure 5.** The two-stage statistical model used in the study. Epigenetic age and pace of epigenetic age was first derived at three time points (age 7, 17, and 24). In the first stage, linear mixed-effects models with chronological age, sex, and cell type composition as predictors and epigenetic age as the outcome generated best linear unbiased predictors (BLUPs), random intercepts and slopes for each individual. In the second stage, these random intercepts and slopes were included in a logistic regression model as predictors with PLE as the outcome.

**SM Results**

|  | **Total (n=1840)** | **Age 7 (n=600)** | **Age 17 (n=1687)** | **Age 24 (n=807)** |
| --- | --- | --- | --- | --- |
| **Age (mean, SD)** | | 7.44 (0.14) | 17.6 (0.70) | 24.4 (0.75) |
| **Biological Sex (n, %)** |  |  |  |  |
| Female | 1046 (56.8%) | 353 (58.8%) | 982 (58.2%) | 397 (49.2%) |
| Male | 794 (43.2%) | 247 (41.2%) | 705 (41.8%) | 410 (50.8%) |
| **DNAm Age (mean, SD)** |  |  |  |  |
| Horvath EA |  | 11.2 (3.60) | 21.7 (4.88) | 27.7 (5.00) |
| Cortical EA |  | 14.1 (2.69) | 27.2 (4.91) | 34.5 (4.15) |
| Dunedin EA |  | 0.84 (0.11) | 0.84 (0.12) | 0.87 (0.12) |
| PCGrimAge EA |  | 25.9 (2.43) | 34.3 (2.62) | 40.1 (2.74) |
| **PLEs at age 24 (n, %)** |  |  |  |  |
| Case | 95 (5.2%) | 26 (4.3%) | 81 (4.8%) | 46 (5.7%) |
| Control | 1745 (94.8%) | 574 (95.7%) | 1606 (95.2%) | 761 (94.3%) |

Supplementary Table 2. Descriptive statistics for the study sample at each time point. EA = epigenetic age, SD = standard deviation.


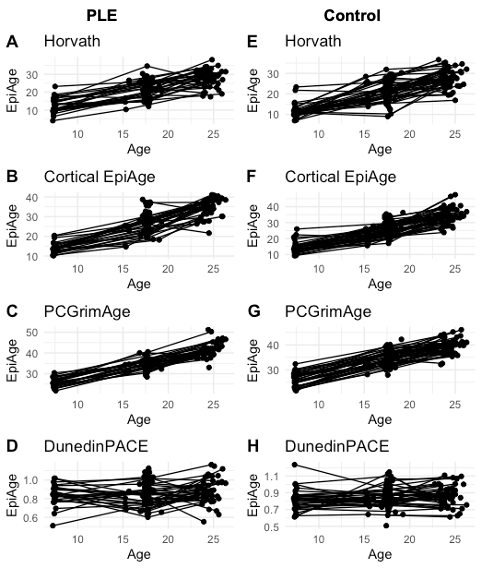


**Supplementary Figure 6.** Spaghetti plots of epigenetic age from age 7 to 24 for individuals with PLEs at age 24 (n = 95) (Panels A-D) and a random sample of 95 controls (Panels E-H).


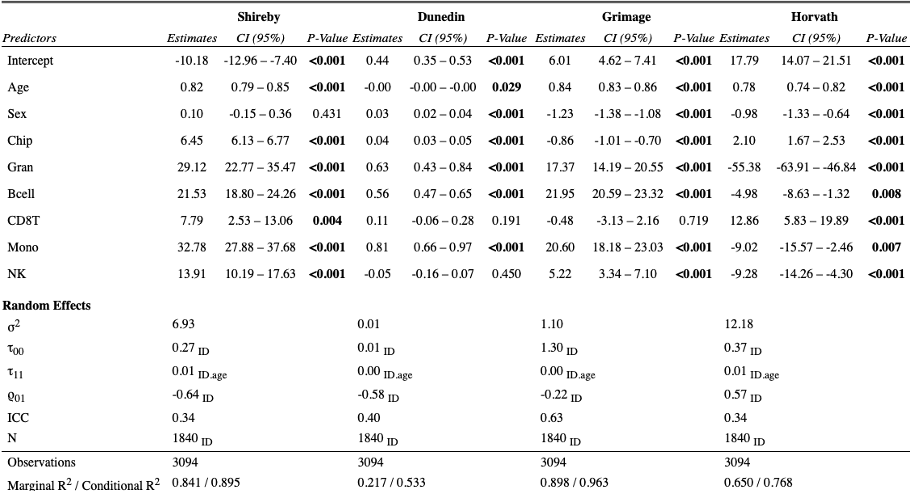


**Supplementary Table 3.** Linear-mixed model output for the primary analysis.


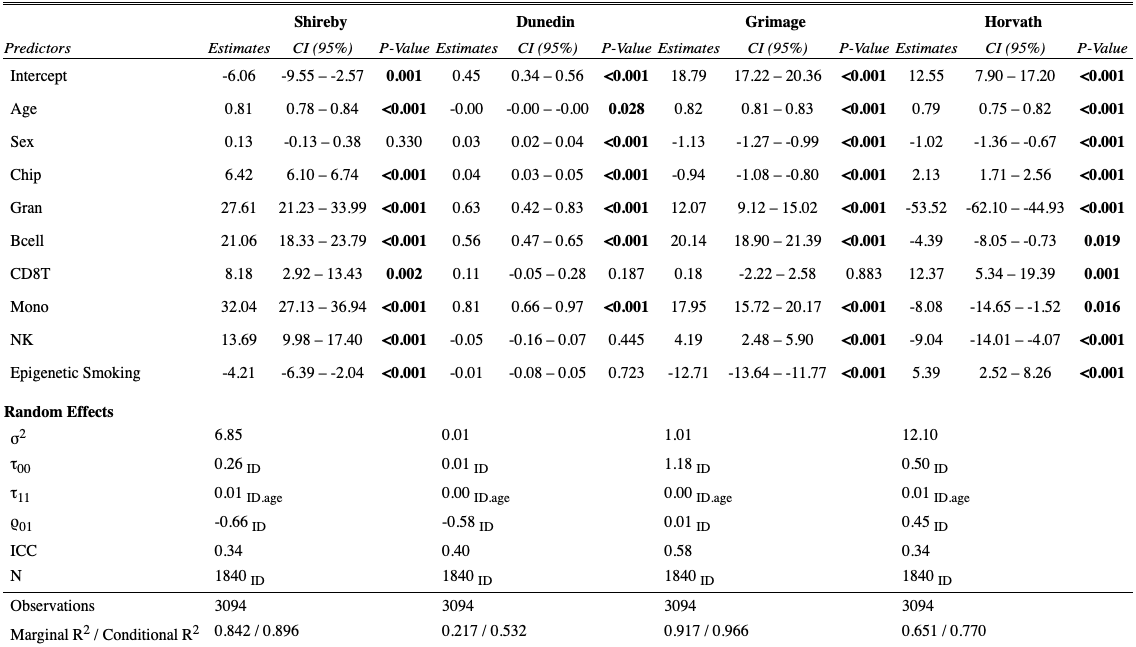


**Supplementary Table 4.** Linear-mixed model output for the secondary analysis.

**References**

Belsky, D. W., Caspi, A., Corcoran, D. L., Sugden, K., Poulton, R., Arseneault, L., … Moffitt, T. E. (2022). DunedinPACE. In *A DNA methylation biomarker of the pace of aging. eLife* (p. 11). doi: 10.7554/elife.73420.

Boyd, A., Golding, J., Macleod, J., Lawlor, D. A., Fraser, A., Henderson, J., … Davey Smith, G. (2013). Cohort Profile: The ‘Children of the 90s’—the index offspring of the Avon Longitudinal Study of Parents and Children. *International Journal of Epidemiology*, *42*(1), 111–127. doi: 10.1093/ije/dys064

Fraser, A., Macdonald-Wallis, C., Tilling, K., Boyd, A., Golding, J., Davey Smith, G., … Ness, A. (2013). Cohort profile: The Avon Longitudinal Study of Parents and Children: ALSPAC mothers cohort. *International Journal of Epidemiology*, *42*(1), 97–110.

Harris, P. A., Taylor, R., Thielke, R., Payne, J., Gonzalez, N., & Conde, J. G. (2009). Research electronic data capture (REDCap)—A metadata-driven methodology and workflow process for providing translational research informatics support. *Journal of Biomedical Informatics*, *42*(2), 377–381. doi: 10.1016/j.jbi.2008.08.010

Horvath, S. (2013). DNA methylation age of human tissues and cell types. *Genome Biology*, *14*(10), R115. doi: 10.1186/gb-2013-14-10-r115

Lu, A. T., Quach, A., Wilson, J. G., Reiner, A. P., Aviv, A., Raj, K., … Horvath, S. (2019). DNA methylation GrimAge strongly predicts lifespan and healthspan. *Aging*, *11*(2), 303–327. doi: 10.18632/aging.101684

Northstone, K., Lewcock, M., Groom, A., Boyd, A., Macleod, J., Timpson, N., & Wells, N. (2019). The Avon Longitudinal Study of Parents and Children (ALSPAC): An update on the enrolled sample of index children in 2019. *Wellcome Open Research*, *4*, 51. doi: 10.12688/wellcomeopenres.15132.1

Relton, C. L., Gaunt, T., McArdle, W., Ho, K., Duggirala, A., Shihab, H., … Davey Smith, G. (2015). Data Resource Profile: Accessible Resource for Integrated Epigenomic Studies (ARIES). *International Journal of Epidemiology*, *44*(4), 1181–1190. doi: 10.1093/ije/dyv072

Shireby, G. L., Davies, J. P., Francis, P. T., Burrage, J., Walker, E. M., Neilson, G. W. A., … Mill, J. (2020). Recalibrating the epigenetic clock: Implications for assessing biological age in the human cortex. *Brain*, *143*(12), 3763–3775. doi: 10.1093/brain/awaa334.
